# Food Transport of Red Imported Fire Ants (Hymenoptera: Formicidae) on Vertical Surfaces

**DOI:** 10.1038/s41598-019-39756-4

**Published:** 2019-03-01

**Authors:** Wenquan Qin, Shucong Lin, Xuan Chen, Jian Chen, Lei Wang, Hongpeng Xiong, Qinxi Xie, Zhaohui Sun, Xiujun Wen, Cai Wang

**Affiliations:** 1 Guangdong Key Laboratory for Innovation Development and Utilization of Forest Plant Germplasm, College of Forestry and Landscape Architecture, South China Agricultural University, Guangzhou, 510642 China; 2 Department of Biology, Salisbury University, Salisbury, MD 21801 USA; 3 Biological Control of Pests Research Unit, Agricultural Research Service, U.S. Department of Agriculture, Stoneville, MS 38776 USA; 4 College of Agriculture, South China Agricultural University, Guangzhou, 510642 China

## Abstract

Many ants can cooperatively transport large food items (either coordinated or uncoordinated during transportation), which can be rarely observed in other animals besides humans. Although these behaviors have been extensively investigated on horizontal surfaces, few studies dealt with food transport on vertical surfaces. The red imported fire ant, *Solenopsis invicta* Buren, is an invasive ant species that commonly forages on trees. Our studies showed that *S. invicta* used multiple strategies to transport food items on vertical surfaces (tree trunks). Small food items (1 × 1 × 1 mm sausage) were carried and transported by individual ants, and larger food items were either collectively and directly transported or cut collaboratively first and small particles were then transported individually or collectively. Competition and deadlocks were frequently observed during individual and collective transport respectively. During cutting, groups of ants tightly fixed the food on the tree trunks by holding the edges of the food item, while other ants cut the food into smaller particles. All food items and particles were moved downward. We investigated the effects of food placement (placed on a platform or fixed on tree trunk), food shape (cuboid or flattened), particle sizes (0.45–1, 1–2, 2–3, or 3–4 mm), and placement height (20, 80, or 150 cm) on the food transport on tree trunks. Our studies are the first to show how fire ants transport food on a vertical surface, and may provide insights into the development of novel fire ant baiting systems that can be placed on tree trunks.

## Introduction

Ants are eusocial insects that perform many collective behaviors for different tasks, such as nest construction, foraging, and defense^1–6^. Among them, behaviors related to foraging processes are critical to the survival of ant colonies. Ants have evolved diverse foraging behaviors to acquire and utilize food in different habitats and environmental conditions. Some aspects of foraging behaviors, such as the cooperative food transport (multiple individuals simultaneously moving large food items), are fairly common in ants but can be rarely observed in other animals besides humans^7,8^.

Previous studies showed that the food transport processes are highly efficient in some ant species. Czaczkes and Ratnieks^7^ reviewed cooperative transport by ants and defined two types of well-coordinated food transport: (1) encircling coordinated transport, and (2) forward-facing coordinated transport. The former type can be observed in ants such as *Leptogenys diminuta* Smith, *Pheidologeton diversus* (Jerdon), *Novomessor cockerelli* (Andre), and some *Pheidole* species^9–11^. During transportation, these ants gather around food items and hold the edge of the food with their mandibles and front legs. The latter type was only reported in the army ants that faced the same direction while transporting food items^12^. For both types, food can be transported fast with few or no deadlocks. Likewise, McCreery and Breed^8^ defined the efficient cooperative food transport in ants as “additional workers arrive at the food quickly, and transport progresses rapidly in a straight line toward the nest”.

Some ants also use the two-way trails to connect nests and food sources. The trails provide navigation information and therefore may enhance the food transport efficiency^13–15^. The forming and maintaining of these “fast-track” paths largely depends on the short-lived but accurate trail pheromones dropped by individual ants on the substance. Importantly, the pheromone deposition by ants is flexible, and therefore the foraging paths can be rapidly adjusted in response to environmental changes^16,17^ and other situations (e.g. trail crowding^18^ and obstacle presence^19^). In addition, the cognition (i.e., memory and learning) of individuals may also play a role in the recruitment and food transportation on trails^20,21^.

Many ants show the behavior of uncoordinated cooperative food transport, characterized by frequently occurring and long-lasting deadlocks^7,8,22^. McCreery and Breed^8^ pointed out that ant species with inefficient transport pattern lack the ability of modifying their behaviors based on other individuals that transported the same food item, and therefore cannot reach a consensus. One example is the red imported fire ants, *Solenopsis invicta* Buren, a significant ecological, agricultural, and medical pest with a worldwide distribution^23^. Wang *et al*.^24^ found that the cooperative food transport of *S. invicta* workers were typically uncoordinated because they “pull/push the food item in multiple directions, leading to a slow process with frequent deadlocks”.

Most studies on ant foraging behaviors were performed on horizontal surfaces (but see Yamamoto *et al*.^25^ and Wojtusiak *et al*.^26^). It is still unclear how food items were moved on a vertical surface. Yamamoto *et al*.^25^ studied the foraging strategies of 44 ant species that belong to 34 genera. They found that most ground-living ant species cooperatively transport large preys without the fragmentation process, whereas the tree-living ants commonly cut large preys and individually transported fragmented pieces to the nests. Interestingly, *S. invicta* not only forage on ground, but also captured preys (arthropods) in the tree canopies^27^. In a previous field study, we observed massive foraging activities (two-way ant trails) of *S. invicta* on tree trunks that transport preys downward to the nests (mounds) in the ground^28^. Although this process should have extra challenges such as gravity, we rarely observed the falling of preys during the transportation, indicating that the food transport by *S. invicta* workers is well organized on vertical surfaces with undetermined behavioral patterns.

Due to the extensive environmental impacts and economic costs associated with *S. invicta*, many studies have focused on the factors that affect food transport process of this pest, which may bring useful insights to develop fire ant baiting technologies. For example, Hooper-Bui *et al*.^29^ reported that *S. invicta* removed significantly more bait particles with sizes >2000 μm as compared to the smaller ones. In our previous work, we developed a novel fire ant baiting system placed on tree trunks^28^. This system is suitable for controlling fire ant populations in urban areas, because regularly clean of the paved surfaces in cities may remove granular baits before they can be found and foraged by fire ants^28^. Enhancing the understanding of vertical food transport behaviors of *S. invicta* would contribute to improve the design of tree trunk-placed baiting systems.

In the present study, we conducted a set of experiments under field condition to answer the following questions: (1) is vertical food transport by *S. invicta* “uncoordinated” like that on a horizontal surface? (2) What are the behavioral patterns and potential adaptations for vertical food transport? And (3) which factors affect the food transport on tree trunks?

## Results

### Behavioral patterns of vertical food transport

**Figure 1:**
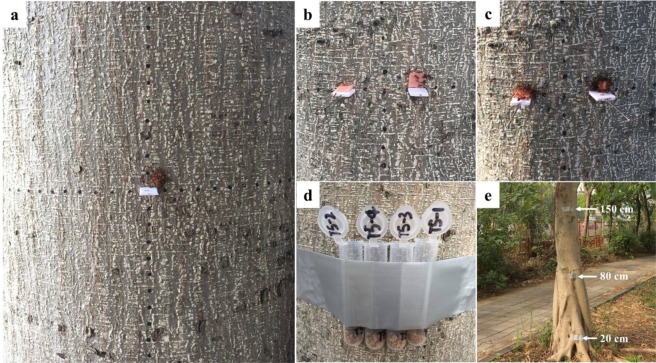
Experimental set-up: (**a**) An observation area (20 × 20 cm) was created on tree trunks by drawing a dashed rectangular (the adjacent dots were 1 cm apart) using a black marker pen, and a food releasing platform (1.0 × 1.0 × 0.2 cm Polyvinyl chloride plate) was fixed in the center of the observation area using an insect pin. (**b**) Two food-releasing platforms were fixed on the tree trunk, and each one was 1.5 cm apart from the center point. A food (8 × 8 × 1 mm sausage) was placed on one food-releasing platforms and contacted with the tree trunk. For the other platform, a food with the same size was fixed on the tree trunk (the bottom side of the food was 2 mm apart from the platform) using an insect pin. (**c**) Two food items with different shapes (cuboid or flattened) were randomly placed on the left or right platform. (**d**) Four tubes containing food particles with different sizes (diameters ranging from 0.45–1, 1–2, 2–3, or 3–4 mm) were attached to tree trunks at the height of ~130 cm. (**e**) Three tubes containing the similar amount of food were attached to tree trunks at the height of 20, 80, or 150 cm.

**Figure 2:**
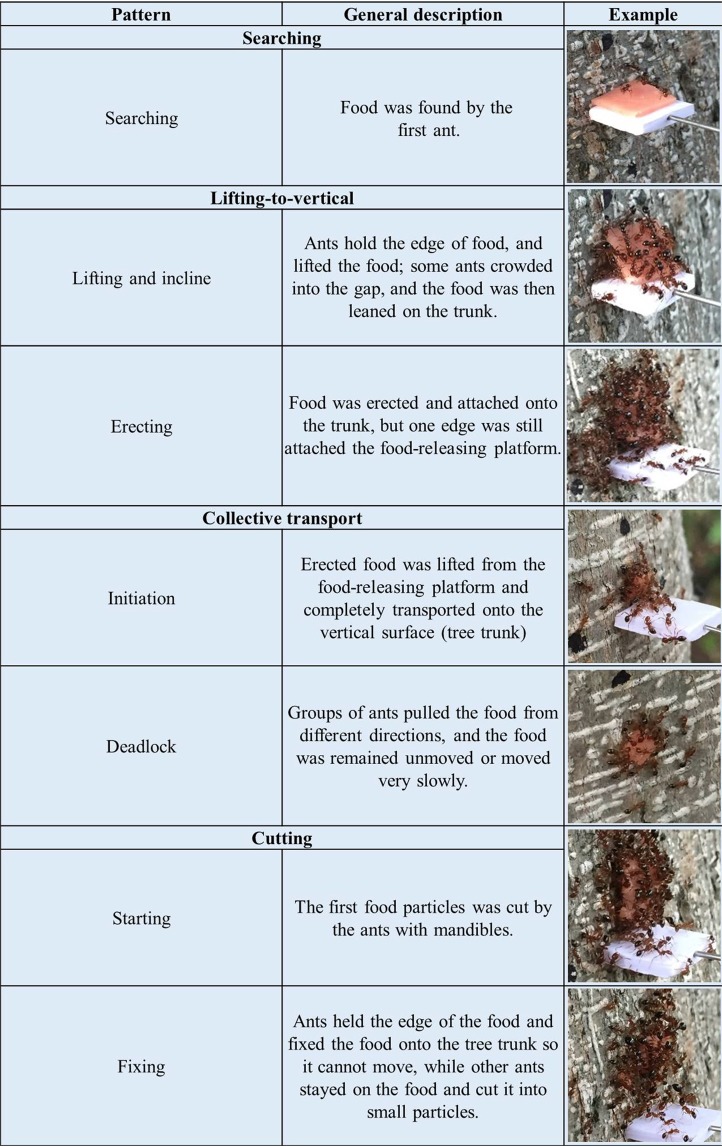
Screen snapshots showing searching, lifting-to-vertical, and transportation (collective transport and cutting) behaviors during vertical food transport by *Solenopsis invicta* workers. Note that the size of food releasing platform (Polyvinyl chloride plate) was 1.0 × 1.0 × 0.2 cm.

This experiment investigated the behavioral patterns and transport efficiency of *S. invicta* workers when they moved food items on tree trunks. We did this by providing food items of various sizes (1 × 1 × 1 mm, 3 × 3 × 1 mm, 5 × 5 × 1 mm, or 8 × 8 × 1 mm sausage) to ants on platforms set up on trees (Fig. 1a). No falling of food was observed during transportation in this experiment. Duration of the searching phase was not significantly different among the four food sizes (Tables 1 and 2). For the small food (1 × 1 × 1 mm), individual ants carried the food and immediately transported it from the releasing platform to the tree trunk. Thus no lifting-to-vertical phase was observed. However, two complicated patterns were observed during the lifting-to-vertical phase for larger food items (3 × 3 × 1 mm, 5 × 5 × 1 mm, or 8 × 8 × 1 mm): (1) lifting and incline: a few ants first lifted one edge of the food item, then some ants squeezed into the gap between the food and platform; as more ants crowded into the gap and pulled the edge of the food, they leaned the sausage on (or against) the tree trunk; and (2) erection: the food item was then parallel and attached to the tree trunks (Fig. 2, Table 1). The two patterns were observed during the lifting-to-vertical phase for almost all large food items (3 × 3 × 1 mm, 5 × 5 × 1 mm, and 8 × 8 × 1 mm) except in one trial (3 × 3 × 1 mm) in which the erection stage was not observed (Food was directly lifted and transported to the vertical surface by ants when it was inclined). Duration of the lifting-to-vertical phase increased with the food size (Tables 1 and 2).

**Table 1:** Time point (min: sec) of behavioral patterns and stages of each phase during vertical food transport by *Solenopsis invicta* workers.

Food size (mm)	No.	Searching phase	Lifting-to-vertical phase		Transportation phase								
					Individual transport			Collective transport			Cutting		
		Searching	Lifting and incline	Erection	Initiation	Competition	End	Initiation	Deadlock	End	Starting	Fixing	End
1 × 1 × 1	1	04:02			04:09		04:39						
	2	01:22			01:48		02:38						
	3	00:08			00:47	00:57	02:46						
	4	03:55			05:39	05:41	09:13						
	5	00:12			04:23		05:20						
	6	00:00			00:25	00:27	01:20						
3 × 3 × 1	1	05:47	18:07					18:08	19:52	32:25			
	2	00:24	00:39	00:47				02:25	02:29	10:51			
	3	05:43	07:18	07:20				10:20	10:22	45:34			
	4	00:37	02:55	06:20				08:13	09:22	25:40			
	5	01:49	11:41	13:03				13:23	13:30	36:33			
	6	02:19	13:18	15:27				16:34	16:37	38:54			
5 × 5 × 1	1	01:27	09:08	15:55							24:19	17:00	
	2	00:15	09:30	12:06							40:54	12:37	63:25
	3	00:07	06:26	07:03							26:20	08:43	46:03
	4	03:41	08:29	09:09							28:27	31:50	
	5	00:00	06:24	16:34							23:09	16:52	
	6	06:29	39:35	50:42				59:34	59:45	64:17			
8 × 8 × 1	1	00:49	16:02	39:12							23:34	46:27	66:26
	2	00:34	10:19	17:52							20:13	19:41	
	3	00:23	09:49	29:13							18:10	37:24	59:51
	4	00:48	11:02	35:30							33:12	45:52	68:33
	5	01:28	15:19	53:09							26:32	67:57	
	6	02:54	27:49	29:29							43:03	42:51

**Table 2:** Duration of different phases, the efficiency of food transport and the percentage of time accounted for when the food remained unmoved by *Solenopsis invicta* workers on the tree trunks.

	1 × 1 × 1 mm	3 × 3 × 1 mm	5 × 5 × 1 mm	8 × 8 × 1 mm	Statistical results
Searching (sec)	96.5 ± 46.5a	179.8 ± 59.4a	119.8 ± 63.7a	69.3 ± 22.8a	*F* = 0.86; *df* = 3, 20; *P* = 0.4764
Lifting-to-vertical (sec)	—	421.0 ± 133.1c	995.0 ± 347.7b	1974.8 ± 286.9a	*F* = 8.38; *df* = 2,15; *P* = 0.0036
Transportation (sec)	162.8 ± 50.1b	1298.7 ± 238.0a	—	—	*t* = −4.67; *df* = 5.4431; *P* = 0.0044
Transport efficiency (g × cm × min^−1^ × worker^−1^)	0.0126 ± 0.0028a	0.0013 ± 0.0002b	—	—	*t* = 4.07; *df* = 5.0675; *P* = 0.0094
Competition, deadlock or fixing (%)	16.6 ± 7.7b	59.0 ± 7.8ab	66.8 ± 10.5a	76.1 ± 7.1a	*χ*^2^ = 12.29; *df* = 3; *P* = 0.0064

Data are presented as mean ± SE. Different letters indicate significant difference (*P* < 0.05).

Three strategies were identified during the transportation phase: (1) individual transport – single ant carried and moved the food item; (2) collective transport – group of ants moved the food item without cutting it into smaller pieces; and (3) cutting – ants cut the food into small particles, which were then transported individually or collectively (Fig. 2; Table 1). For the collective transport, the whole part of the food was transported outside the observation area, which can be exclusively distinguished from cutting. All small food items (1 × 1 × 1 mm) were individually transported, and all medium-sized food items (3 × 3 × 1 mm) and one larger food (5 × 5 × 1 mm) were collectively transported, whereas all remaining food items were cut into small pieces (Table 1).

**Figure 3:**
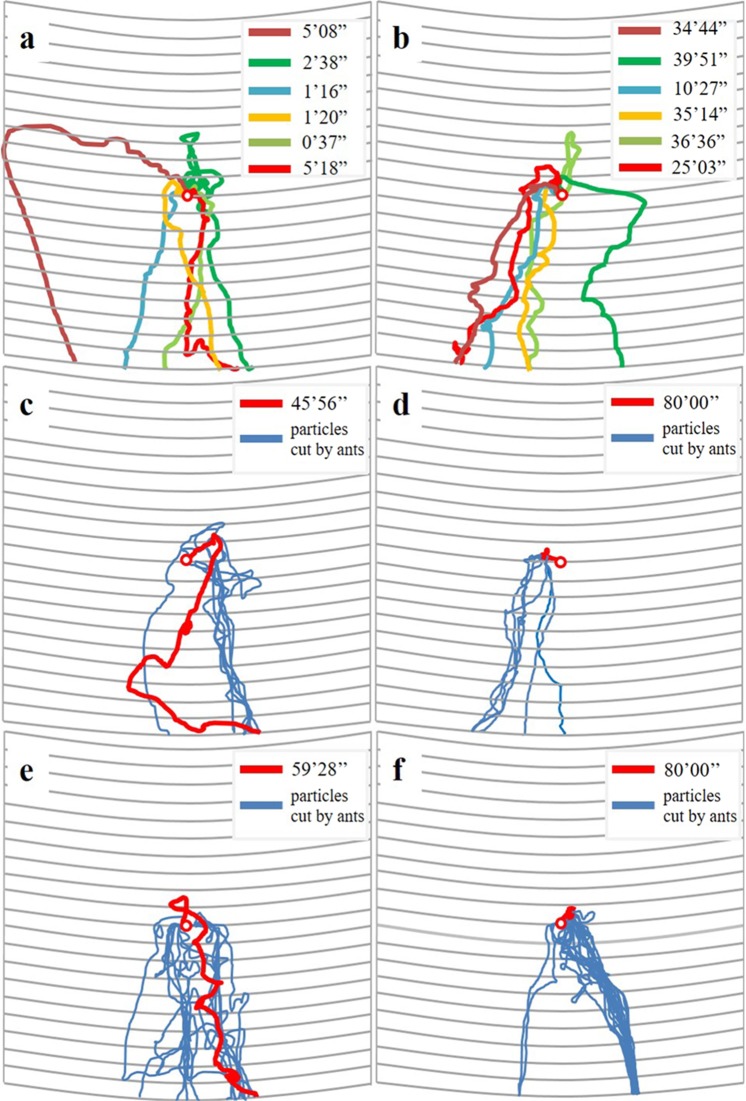
The transport paths of food of different sizes – i.e., (**a**) 1 × 1 × 1 mm, (**b**) 3 × 3 × 1 mm, (**c,d**) 5 × 5 × 1 mm, and (**e,f**) 8 × 8 × 1 mm – traced on the observation area on tree trunks. For (**a**) 1 × 1 × 1 and (**b**) 3 × 3 × 1 mm food, different colors indicate the transport path of the six replicates. For (**c,d**) 5 × 5 × 1 and (**e,f**) 8 × 8 × 1 mm food, the transport path of 1 replicate was shown, with red lines indicating the path of main part of the food item, and blue lines indicating the paths of particles cut by ants. Note that during cutting, the main part of 5 × 5 × 1 or 8 × 8 × 1 mm food was either slowly moved (**c,e**), or remained unmoved during the 80-min observation period (**d,f**). The red circle indicates the location where the food was initially placed. The bars followed by numbers indicate the time between food was searched and transported (individually or collectively) outside the observation area (**a,b,c,e**), or until 80 min had passed after food releasing (**d,f**). The distance between each adjacent scale lines indicates 1 cm apart.

All food items were first transported upward for 1–40 mm, and then downward to the ground (Fig. 3a–f). During individual transport of small food, we observed competition behavior (two ants grabbing the same food) in 3 of the 6 videos (Table 1). And in each case of competition one ant finally took the food and continued to transport the food individually. In all videos of the collective transport, groups of ants (more than two individuals) frequently pulled the food in different directions, causing deadlocks in which food remained unmoved or moved slowly (Fig. 2; Table 1). During cutting, the food was tightly fixed (edge of the food was held by groups of ants) on tree trunks, and the main part of food items (5 × 5 × 1 mm, or 8 × 8 × 1 mm) was either slowly moved (Fig. 3c,e) or remained unmoved during the 80-min observation period (Fig. 3d,f).

The efficiency of individual transport was significantly higher than that of collective transport (Table 2). The percentage of time used for competition during the individual transport was significantly lower than that for fixing during cutting, but both were not significantly different from the percentage of time for deadlocks during collective transport (Table 2).

### Effect of food placement on vertical food transport

**Figure 4:**
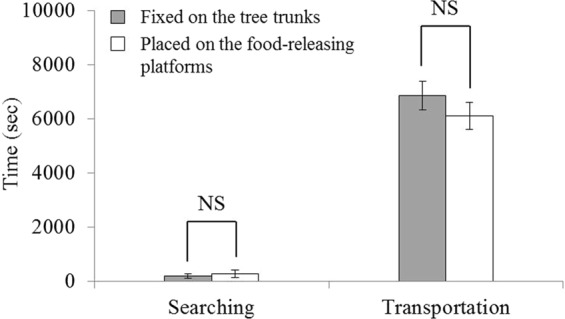
Duration of the searching and transportation phases of food (8 × 8 × 1 mm) that was placed on the food-releasing platform, or directly fixed on the tree trunks with an insect pin. Data are presented as mean ± SE. NS indicates no significant difference (*P* > 0.05). Note that all food items were cut during the transportation phase.

This experiment tested if the food placement affects the pattern and duration of vertical food transport by *S. invicta* workers. Large food items (8 × 8 × 1 mm sausage) were either placed on the platform or artificially fixed on trees (Fig. 1b). We observed that these food items were cut by ants regardless of placement. Durations of the searching phase (*t* = −1.07, *df* = 7, *P* = 0.3212), and the transportation (cutting) phases (*t* = 1.33, *df* = 7, *P* = 0.2247) were not significantly different between the two placements (Fig. 4).

### Effect of food shape on vertical food transport

**Figure 5:**
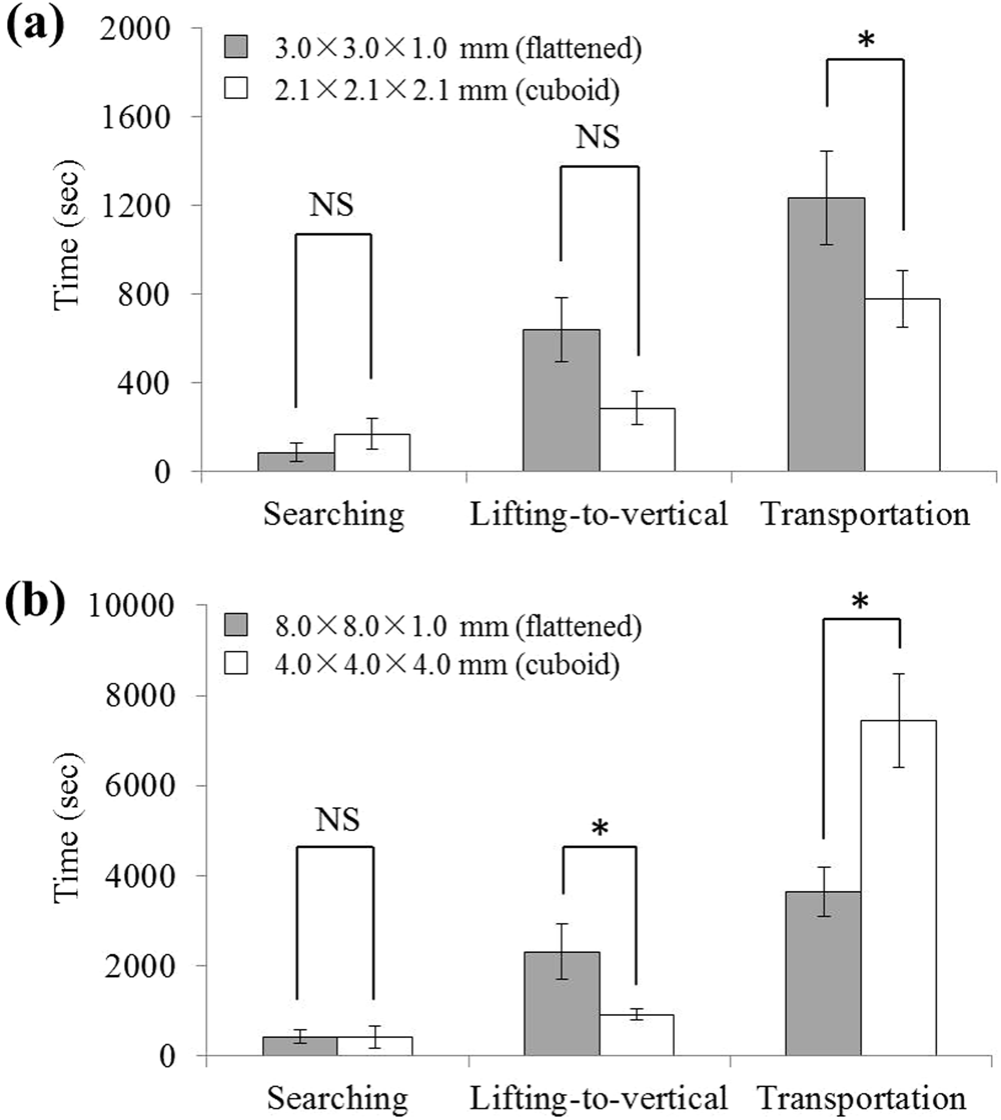
Duration of the searching, lifting-to-vertical and transportation phases of (**a**) medium-sized food (9 mm^3^), and (**b**) large-sized food (64 mm^3^) with different shapes (cuboid or flattened). Data are presented as mean ± SE. The asterisk (*) indicates significant difference (*P* < 0.05), and NS indicates no significant difference (*P* > 0.05).

This experiment tested if the shape of food items affect the pattern and duration of vertical food transport by *S. invicta* workers. We did this by providing the cuboid and flattened food on food-releasing platforms set up on trees (Fig. 1c). In collective transport of medium-sized food (9 mm^3^), durations of the searching (*t* = −1.31, *df* = 8, *P* = 0.2257; Fig. 5a) and lifting-to-vertical phases (*t* = 1.8, *df* = 8, *P* = 0.1092; Fig. 5a) were similar between cuboid (2.1 × 2.1 × 2.1 mm) and flattened (3 × 3 × 1 mm) food. However, ants spent significantly shorter time to transport the cuboid food compared with flattened ones (*t* = 2.49, *df* = 8, *P* = 0.0377; Fig. 5a).

The large-sized food (64 mm^3^) were cut during the transportation phase. The duration of searching phase was not significantly different between cuboid (4 × 4 × 4 mm) and flattened (8 × 8 × 1 mm) food (*t* = 0.02, *df* = 8, *P* = 0.9870; Fig. 5b). However, ants spent significantly less time in moving cuboid food during the lifting-to-vertical phase (*t* = 2.56, *df* = 8, *P* = 0.0338) but more time during the transportation phase (*t* = −3.63, *df* = 8, *P* = 0.0067) (Fig. 5b).

### Effect of particle size on vertical food transport

**Figure 6:**
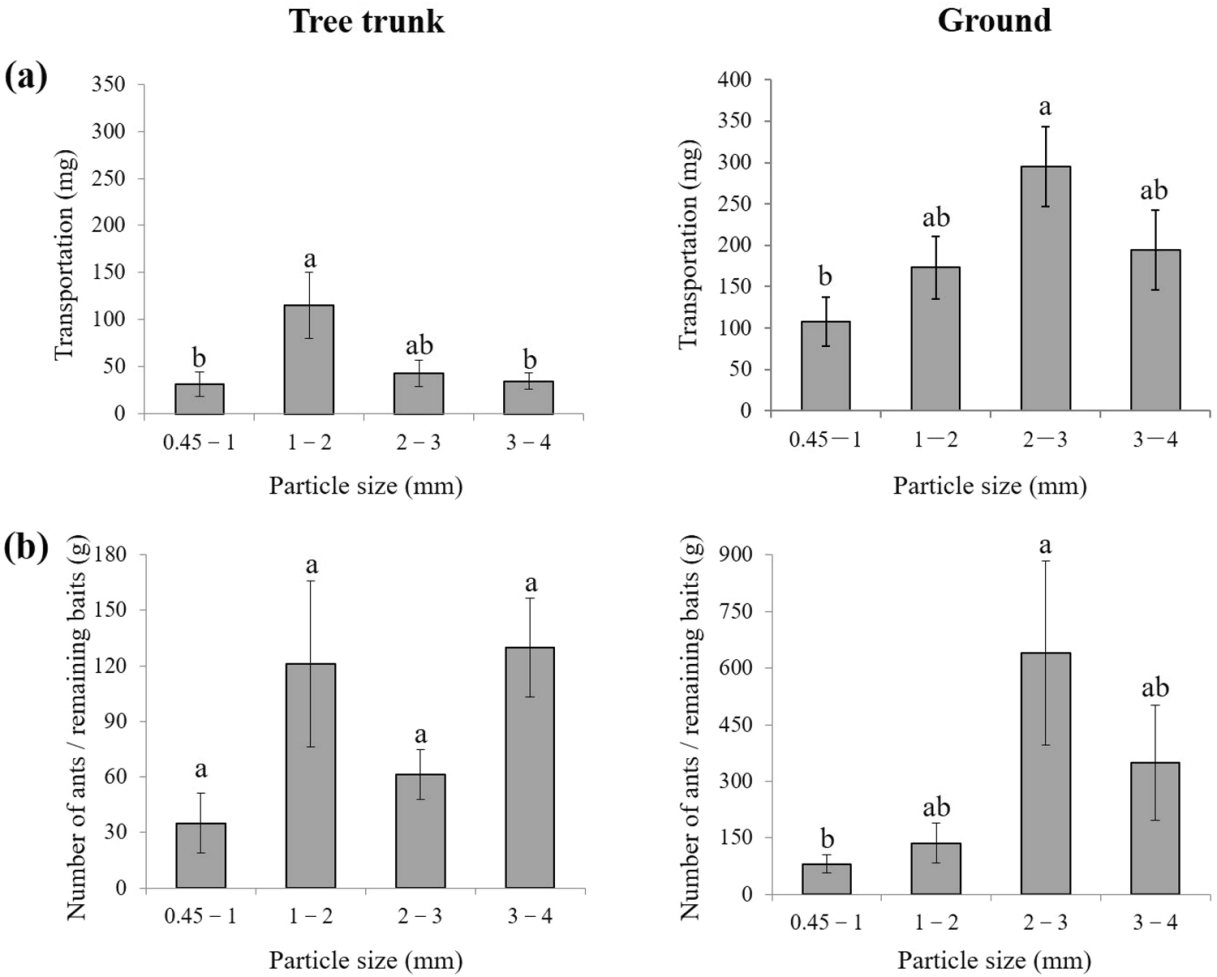
(**a**) The food transportation (weight change of food before and after the experiment) and (**b**) the number of ants per gram of the remaining food in each tube containing food particle sizes (diameters) ranging from 0.45–1, 1–2, 2–3, or 3–4 mm. These tubes were attached to tree trunks at the height of ~130 cm, or on the ground near each tree (0.8–1.0 m from the root). Data are presented as mean ± SE. Different letters indicate significant difference (*P* < 0.05).

This experiment tested the effect of particle size on food transportation and ant recruitment on vertical and horizontal surfaces. We did this by attaching four tubes containing food particles with different sizes (diameters ranging from 0.45–1, 1–2, 2–3, or 3–4 mm) on tree trunks (Fig. 1d) and on ground. On tree trunks, ants transported significantly more food particles ranging from 1–2 mm than food particles ranging from 0.45–1 mm or 3–4 mm (*F* = 3.78, *df* = 3, 52; *P* = 0.0158; Fig. 6a); however, the number of ants per gram of food remaining in tubes was not significantly different among the four food particle sizes (*F* = 2.66, *df* = 3, 52; *P* = 0.0580; Fig. 6b). On the ground, significantly more food transportation (*F* = 3.42, *df* = 3, 52; *P* = 0.0237) and ants per gram of remaining food (*F* = 2.99, *df* = 3, 52; *P* = 0.0394) were found in the tubes with particles ranging from 2–3 mm than in tubes containing the smallest particles (Fig. 6a,b).

### Effect of Placement Height on Vertical Food Transport

**Figure 7:**
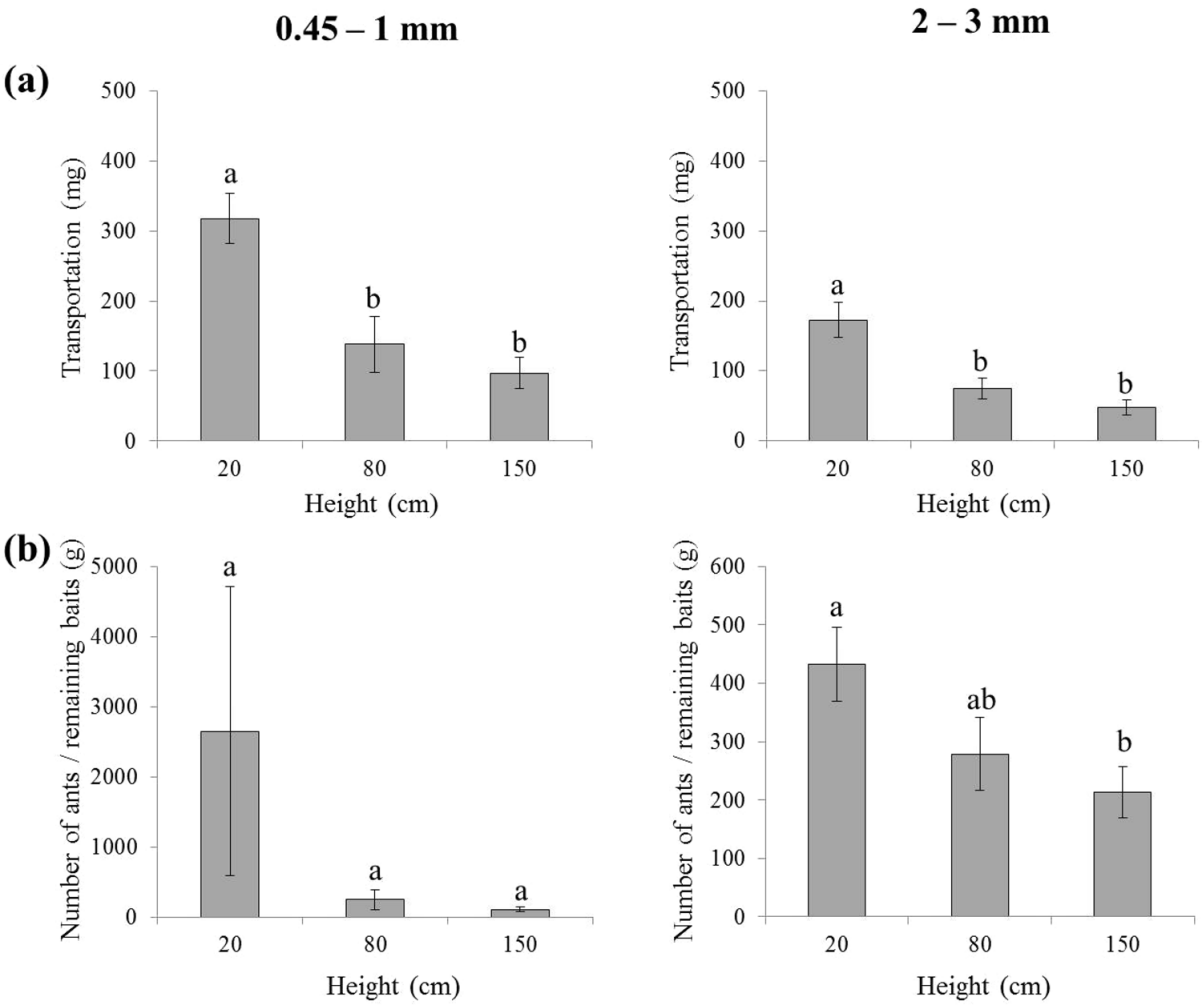
(**a**) The food transportation (weight change of food before and after the experiment) and (**b**) the number of ants per gram of the remaining food in each tube placed on the tree trunks at the height of 20, 80, or 150 cm. Tubes with the particles sizes ranging from 0.45–1 or 2–3 mm were tested separately. Data are presented as mean ± SE. Different letters indicate significant difference (*P* < 0.05).

This experiment tested the effect of placement height on food transportation and ant recruitment on tree trunks. Three tubes containing the similar amount of food were attached to tree trunks at the height of 20, 80, and 150 cm (Fig. 1e). For both particle sizes (0.45–1 or 2–3 mm), significantly more food placed at the height of 20 cm was transported, compared to those at 80 and 150 cm (0.45–1 mm: *F* = 12.28, *df* = 2, 27; *P* = 0.0002; 2–3 mm: *F* = 13.33, *df* = 2, 33; *P* < 0.0001; Fig. 7a). The transportation was similar between foods at the heights of 80 and 150 cm (Fig. 7a). For food with small particle sizes (0.45–1 mm), there were similar number of ants per gram of remaining food in tubes placed at different heights (*F* = 1.43, *df* = 2, 27; *P* = 0.2536; Fig. 7b). However, for larger food particles (2–3 mm), significantly more ants per gram of remaining food were found in the tubes placed at the height of 20 cm than that at 150 cm (*F* = 3.83, *df* = 2, 33; *P* = 0.0319; Fig. 7b).

## Discussion

Although *S. invicta* is one of the most extensively studied ant species, many aspects of its foraging behaviors have been studied just in recent years^24,30,31^. This study is the first to investigate how fire ants transport food items on the vertical surface. Results showed that multiple strategies (individual transport, collective transport or cutting) were used by *S. invicta* workers in response to different food sizes during vertical transportation, and food shape, particle size, and placement height had significant effects on the vertical food transport.

Anderson *et al*.^32^ defined the self-assemblages of eusocial insects as the “physical structures comprised of individuals that have linked themselves to one another”. These structures played critical roles during foraging processes. For example, Peeters and De Greef ^33^ observed that some *Leptogenys* ant species linked their bodies in chains and walked backwards to drag large prey items such as millipedes. The self-assembling in *S. invicta* for foraging purpose was also reported in several recent studies. For example, Wang *et al*.^30^ reported that *S. invicta* workers could cover and feed upon the droplet of liquid food (sucrose water). In the present study, we found that *S. invicta* workers filled the gaps between food and tree trunks. We speculate that these ants may act like a smooth slope by linking the sticky tarsal pads to each other, as they did when forming other self-assembling structures such as rafts and sinking towers^34–36^. By so doing, the food item can be relatively easily pulled/dragged by other ants from the horizontal surface to the vertical surface. It is also possible that ants simply get under the food accidentally in an effort to access it.

Although fire ants exhibit similar behaviors (e.g. competition, deadlocks) when moving food items on horizontal and vertical surfaces, differences were detected between these two transportation conditions. For example, the efficiency of individual and collective food transport of *S. invicta* was not significantly different on the horizontal surface^24^. In the present study, however, the efficiency of individual food transport was much higher than that of collective transport on tree trunks (Table 2). This low-efficiency of the collective vertical food transport is due to the long-lasting deadlocks. McCreery *et al*.^37^ reported that “groups break deadlocks only if individuals more readily give up when they are going against the majority”. It is worth noting that the vertical food transport can be challenged by the gravity, which is likely not a dominating factor during horizontal food transport. Ant on a vertical surface needed to hold the edge of food items, and they may not easily give up their task and adjust transport directions. Upsetting of the appropriate balance among gravity, friction, and dragging forces of ants may either break deadlocks or cause ants to drop food items from the tree. In the present study, we did not observe food-falling events. Ants may prevent food falling by carefully adjusting their position and slowly moving the food items, which may reduce the transport efficiency.

On the ground, food cutting frequently occurs when food items were too big for fire ants to move^24,38,39^. In these previous studies, ants stayed on top of food items and cut the food into small particles with their mandibles. In the present study, many ants stayed around the food items and held the edge of the food with their mandibles and front legs. Thus, the food was tightly fixed on the tree trunks (Supplementary Fig. S1). Although these fixing ants were not directly involved in cutting food, they played an important role in keeping the food on the tree so that other ants could carry out the cutting task. Interestingly, ants preferred to cut the food on the vertical surface instead of on the horizontal food-release platform. Perhaps in the latter case, ants needed to frequently switch between horizontal and vertical surfaces, thus decreasing transport efficiency. It is still unclear why *S. invicta* workers cut the large food items rather than collectively transport them. But the complicated behavioral patterns of ants usually “emerge from groups of individuals obeying relatively simple rules”^37^. Perhaps ants are not trying to cut the food up as long as the item is moving, and only do so when transport is harder on the vertical surface. Our studies also showed that duration of whole cutting process was not significantly different whether the food was placed on the horizontal platform or directly fixed on tree trunks (Fig. 4). In the latter case, the lifting-to-vertical phase (transferring the food from the horizontal platform to the tree trunk) is lacking. It is probable that ants can carry out food-lifting and cutting simultaneously with limited interference between these two tasks.

Our study showed that the shape affected food transportation. For medium-sized food items, ants spent significantly less time collectively transporting the cuboid food (2.1 × 2.1 × 2.1 mm) compared to the flattened one (3.0 × 3.0 × 1.0 mm) (Fig. 5a). In this study, we did not take videos to determine the duration of deadlocks and the number of ant transporters. As a result, it is not known which behavior resulted in the difference. We speculate that flattened food had longer deadlocks, probably because more ants could hold the edge of the flattened food (flattened food has longer edges than that of the cuboid food) during the transport, and therefore makes it more difficult to break the deadlocks as we discussed earlier. Likewise, Buffin *et al*.^40^ reported that the higher group size of *Novomessor cockerelli* (Andre) transporters caused lower food-moving speeds. However, contrasting results were reported in different ant species such as *Paratrechina longicornis* (Latreille), which showed higher speeds for larger group of transporters distributed around the food^41^. Interestingly, the opposite pattern was found for large-sized food, since ants spent significantly more time to cut the cuboid (4.0 × 4.0 × 4.0 mm) food compared to the flattened (8.0 × 8.0 × 1.0 mm) one (Fig. 5b). Perhaps the food edges might be the easiest area for cutting and flattened food have much longer edge than the cuboid one.

*S. invicta* workers transported more food with the particle sizes ranging from 2–3 mm on the ground, which corroborates previous findings^29^. However, ants transported significantly more particles ranging from 1–2 mm on tree trunks (Fig. 6). Perhaps it is more difficult for ants to hold the large particles and move on vertical surfaces as compared to horizontal ones. Taylor^42^ reported that food sources near the nest recruited more *Solenopsis geminate* Fabricius workers compared to food that was more distant to the nests. Similarly, baiting tubes placed near the base of trees attracted more *S. invicta* workers (per gram of remaining food in tubes), which removed more food mass regardless of particle sizes (Fig. 7). These results suggested that using bait particles ranging from 1–2 mm and placing baiting tubes near the base of trees may improve bait removal rate by fire ants.

## Materials and Methods

### Study Site

The field studies were conducted from 25 October 2017 to 18 November 2017 in a green belt (23°09′N, 113°21′E) along the street close to the campus of South China Agricultural University, Guangzhou, China. Our previous studies showed massive *S. invicta* activities on trees (*Ficus concinna* Miquel) in this area^28^. All studies were conducted during the daytime (9:30 am to 6:00 pm), with temperature ranging from 23–31 °C, and relative humidity 30–74%.

### Behavioral observation of vertical food transport

Six *F. concinna* trees with ant trails on trunks were used for this study (Supplementary Table S1). On the trunk of each tree, an observation area (20 × 20 cm) was created by drawing a dashed rectangular (the adjacent dots were 1 cm apart) using a black marker pen (Fig. 1a). A food releasing platform (1.0 × 1.0 × 0.2 cm Polyvinyl chloride plate) was fixed in the center of the observation area using an insect pin (Fig. 1a). A given size of sausage (i.e., 1 × 1 × 1 mm, 3 × 3 × 1 mm, 5 × 5 × 1 mm, or 8 × 8 × 1 mm) was place on the food releasing platform and contacted with the tree trunk. Responses of ants were videotaped until the sausage was transported outside the observation area, or until 80 min after food releasing. For each food size, there was only one trial per tree. Here we did not repeat trials of the same food size on the same tree trunk to avoid pseudoreplication. However, it is important to note that ants from different tree trunks may come from the same colony. In addition, only one video was taken for each tree trunk per day (Supplementary Table S1). The video was replayed and examined in the lab and the vertical food transport process was divided into three phases (Fig. 2): (i) searching phase – starting from the time of food placement until it was detected by the first ant; (ii) lifting-to-vertical phase – from the end of searching phase till the food was moved to the vertical surface (tree trunks); and (iii) transportation phase – from the end of lifting-to-vertical phase till the food was transported (individually or collectively) outside the observation area as one whole piece, or cut into small particles which were then transported outside the observation area individually or collectively by ants.

The food transport patterns (strategies) in response to different food sizes were identified and described for each phase. The transport path of the food (as well as the food particles cut by ants) was traced by recoding the location of individually transported food (see results) in every 2 s, and collectively transported food in every 30 s. For the food items cut by ants, the location of main part of the food was recorded in every 1 minute, and the location of cut particles was recorded in every 2 s (for individually transported particles) or 30 s (for collective transported particles). No location information was recorded during deadlocks and fixing (see results) when the food (or main part of the food) was unmoved or moved very slowly. During the transportation phase, the percentage of time when food was not moved (i.e., competition between individuals, deadlocks due to collective transport, or fixing during cutting) was calculated. For individual and collective transport, the transported efficiency was calculated using the formula provided by McCreery and Breed^8^:

$$\mathrm{Transport}\,\mathrm{efficiency}=\tfrac{\mathrm{mass}\,\mathrm{of}\,\mathrm{food}\times \mathrm{straightline}\,\mathrm{distance}\,\mathrm{between}\,\mathrm{the}\,\mathrm{starting}\,\mathrm{to}\,\mathrm{ending}\,\mathrm{point}\,\mathrm{of}\,\mathrm{food}\,\mathrm{transport}}{\mathrm{number}\,\mathrm{of}\,\mathrm{workers}\,\mathrm{in}\,\mathrm{the}\,\mathrm{transport}\,\mathrm{group}\times \mathrm{time}\,\mathrm{for}\,\mathrm{food}\,\mathrm{transport}}$$

Because some ants joined or left the food transport group over time, we counted the number of ant transporters in every 5 min across the transport process and then averaged. Transport efficiency as well as the time duration for each phase were compared among different food sizes using the *t*-test (Proc ttest, SAS 9.4, SAS Institute, Cary, NC), or one-way analysis of variance (ANOVA, Proc Mixed, SAS 9.4), followed by Tukey’s Honest Significant Differences (HSD) tests for multiple comparisons. Because the percentage of time accounting for the food that remaining unmoved were not normally distributed, these data were compared among different food sizes using the nonparametric Kruskal–Wallis test followed by Dunn’s post-hoc test for multiple comparisons^43^.

### Effect of food placement on vertical food transport

Because *S. invicta* workers need to transport and fix the large food items on tree trunks for cutting (see results), we conducted a study to investigate whether the food placement (placed on the food-releasing platform or artificially fixed on the tree trunk) affect the vertical food transport of ants. Eight *F. concinna* trees with ant trails on the trunks were used for this study (only one trial was conducted per tree). An observation area was created using the same method as described above. Two food-releasing platforms were fixed on the tree trunk, and each one was 1.5 cm apart from the center point (Fig. 1b). A food (8 × 8 × 1 mm sausage) was placed on one food-releasing platform. Each food item was in contact with the tree trunk. For the other platform, a food item with the same size was fixed on the tree trunk (the bottom side of the food was 2 mm apart from the platform) using an insect pin (Fig. 1b). The pins were not removed throughout the experiment to avoid to disturb foraging ants. The weight of each of the two food items (placed on the food-releasing platform or fixed on the tree trunk) was similar (Supplementary Fig. S2a), and the releasing mode was randomly assigned to the left or right of the platform. The time durations between food placing and discovering (searching phase), and between food discovering and completely cutting (lifting-to-vertical and transportation phases), were recoded and compared using the paired *t*-test (Proc ttest, SAS 9.4).

### Effect of food shape on vertical food transport

Nine *F. concinna* trees were selected to investigate the effect of food shape on transport behavior on tree trunks. The two food-releasing platforms were prepared as mentioned above. Two medium-sized food items with different shapes – cuboid (2.1 × 2.1 × 2.1 mm) or flattened (3.0 × 3.0 × 1.0 mm) – were randomly placed on either left or the right platform (Fig. 1c). We also repeated the experiment using large-sized food items (cuboid food: 4.0 × 4.0 × 4.0 mm, or flattened food: 8.0 × 8.0 × 1.0 mm). For each size, the weight of the cuboid or flattened food was similar (Supplementary Material: Fig. S2b,c). The trials of the same food size were not repeated on the same tree trunk, and only one test was conducted each day for each tree. Time durations of searching, lifting-to-vertical, and transportation phases were recorded and compared using the paired *t*-test (Proc ttest, SAS 9.4).

### Effect of particle size on vertical food transport

Fourteen *F. concinna* trees were selected to investigate the effect of particle size on the food transport by *S. invicta* workers on tree trunks (only one trial was conducted per tree). Dog food (Royal Canin^®^, Shanghai, China) was ground with pestles and mortars, then sifted through 4-, 3-, 2-, 1-, and 0.45-mm sieves. As a result, the food particles with sizes (diameters) ranging from 0.45–1, 1–2, 2–3, or 3–4 mm were obtained. Approximately 500 mg food (Supplementary Fig. S3) of each size was weighed using a 0.1 mg electronic balance and added into a 10-mL Eppendorf tube. Four tubes containing food particles with different sizes were attached to tree trunks using sticky tapes (V-Tech^®^, Guangdong, China) at the height of ~130 cm (Fig. 1d). To compare the food transport on vertical and horizontal surfaces, the same set of tubes were also attached to the ground near each tree (0.8–1.0 m from the root). The order of tubes was randomly assigned. In total, 112 tubes were released. Because food transport on ground was much faster than that on tree trunks, the tubes placed on ground were collected at 1.0–1.5 h after releasing, whereas 3.0–3.5 h on tree trunks. The collected tubes were sealed and brought to the lab and stored in a −20 °C freezer. The ants trapped in each tube was counted, and the food remaining in each tube was weighed using a 0.1 mg electronic balance. The food transportation (weight change of food before and after the experiment) was compared among particle sizes using the one-way ANOVA (Proc Mixed, SAS 9.4). We also evaluated ant recruitment by calculating a ratio of ant number to remaining food in the vial (using the absolute number of ants may cause some bias because the number of recruited ants would decrease after many ants carried the food particles and left), which was then compared among particle sizes using the one-way ANOVA (Proc Mixed, SAS 9.4).

### Effect of placing height on vertical food transport

All food items were transported down the tree (see results), and we hypothesize that ants transport more food at the lower part of the tree. Twelve *F. concinna* trees were selected for this study, and grounded dog food with the particles sizes ranging from 0.45–1 or 2–3 mm was tested separately (only one particle size was tested each day for each tree, and the trials of the same particle size were not repeated on the same tree trunk). For each particle size, three tubes containing the similar amount of food (~500 mg, Supplementary Fig. S4) were attached to tree trunks at the height of 20, 80, or 150 cm (Fig. 1e). The tubes were collected at 2–2.5 h after placement. The food transportation as well as the number of ants per gram of the remaining food were compared among heights using one-way ANOVAs (Proc Mixed, SAS 9.4).

## Supplementary information

Supplementary materials

## Data Availability

The datasets generated and analyzed during the current study are available from the corresponding author on reasonable request.
