# Supplementary material for: Food Transport of Red Imported Fire Ants (Hymenoptera: Formicidae) on Vertical Surfaces
Source: Sci Rep. 2019 Mar 1;9:3283. doi: 10.1038/s41598-019-39756-4 (PMC6397150; doi:10.1038/s41598-019-39756-4)
Supplement: Supplementary file 1 — Supplementary materials [file 41598_2019_39756_MOESM1_ESM.docx]

**Food Transport of Red Imported Fire Ants (Hymenoptera: Formicidae) on Vertical Surfaces**

Wenquan Qin^1,^ **^†^**, Shucong Lin^1,^ **^†^**, Xuan Chen^2^, Jian Chen^3^, Lei Wang^4^,

Hongpeng Xiong^1^, Qinxi Xie^1^, Zhaohui Sun^1^, Xiujun Wen^1,*^, Cai Wang^1,*^

**Affiliations**

^1^ Guangdong Key Laboratory for Innovation Development and Utilization of Forest Plant Germplasm, College of Forestry and Landscape Architecture, South China Agricultural University, Guangzhou, 510642 China. ^2^ Department of Biology, Salisbury University, Salisbury, MD 21801, USA. ^3^ Biological Control of Pests Research Unit, Agricultural Research Service, U.S. Department of Agriculture, Stoneville, MS 38776, USA. ^4^ College of Agriculture, South China Agricultural University, Guangzhou 510642, China.

^†^ These authors contributed equally to this work.

*Correspondence and requests for materials should be addressed to X. W. (e-mail: wenxiujun@scau.edu.cn), or C. W. (e-mail: wangcai@scau.edu.cn)


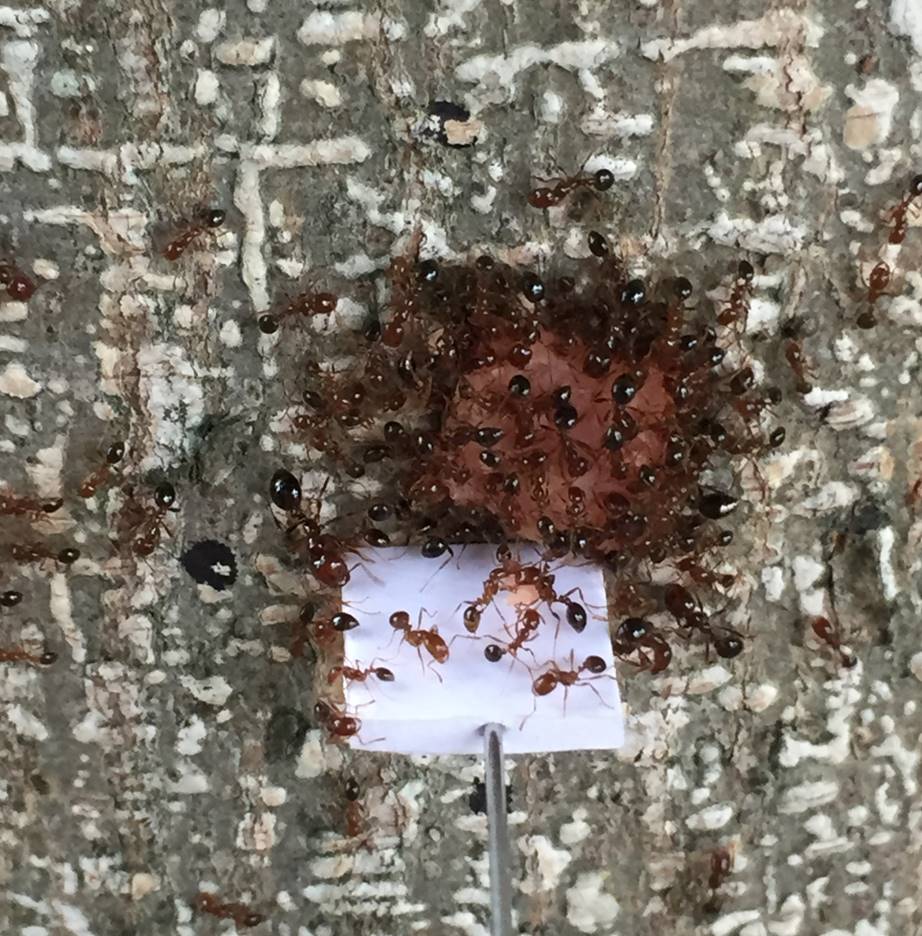


**Fig. S1:** During the food cutting on the vertical surface, many ants stayed around the food items and held the edge of the food with their mandibles and front legs. Thus the food was tightly fixed on the tree trunks. Note that the size of food releasing platform (Polyvinyl chloride plate) was 1.0×1.0×0.2 cm.


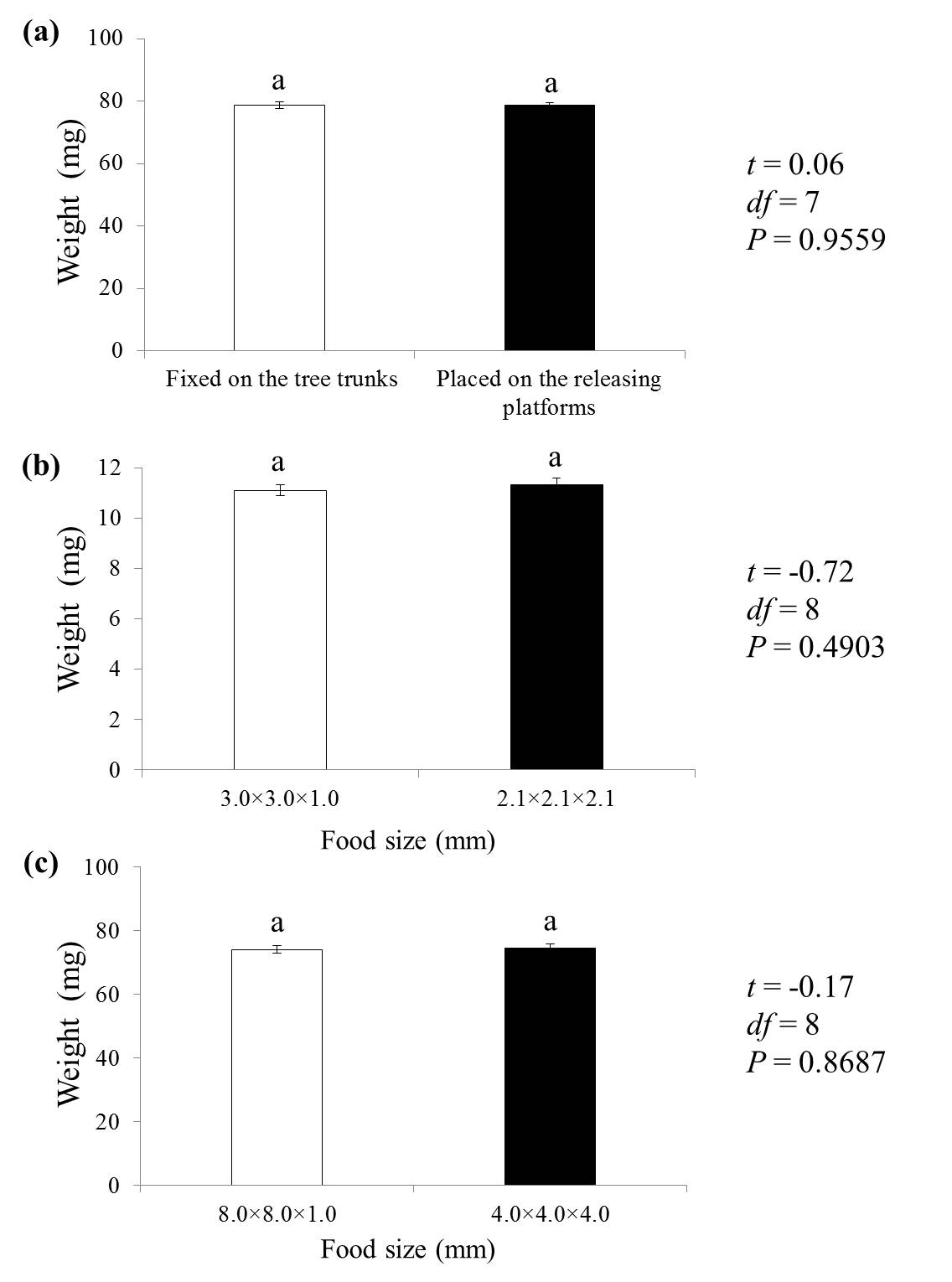


**Fig. S2:** (a) The weight of the food items that were initially placed on the food-releasing platform or fixed on the tree trunk was similar. For (b) medium-sized food or (c) large-sized food, the weight of cuboid or flattened food (randomly placed on the left or right platform) was similar. Data were compared using the paired *t*-test. The same letter indicates no significant difference (*P* > 0.05).


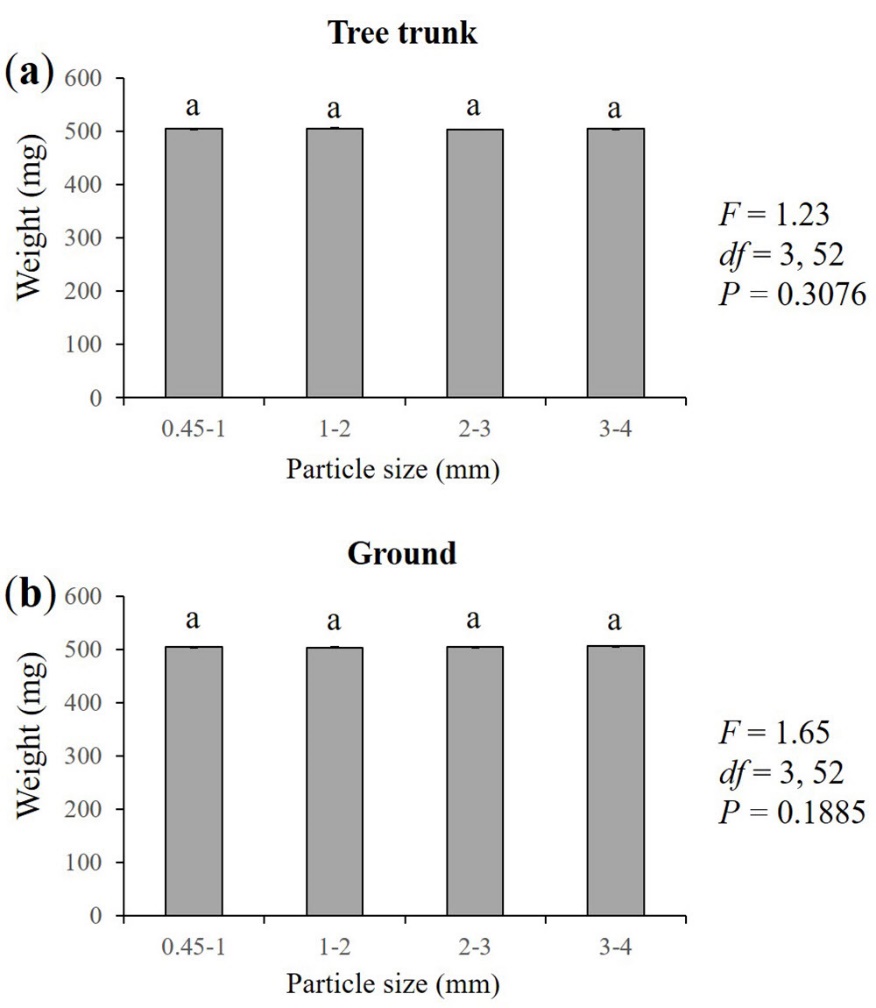


**Fig. S3:** Tubes pasted (a) on the tree trunks or (b) on grounds contained similar weight of food with different particle sizes (0.45-1, 1-2, 2-3, or 3-4 mm). Data were compared using ANOVA. The same letter indicates no significant difference (*P* > 0.05).


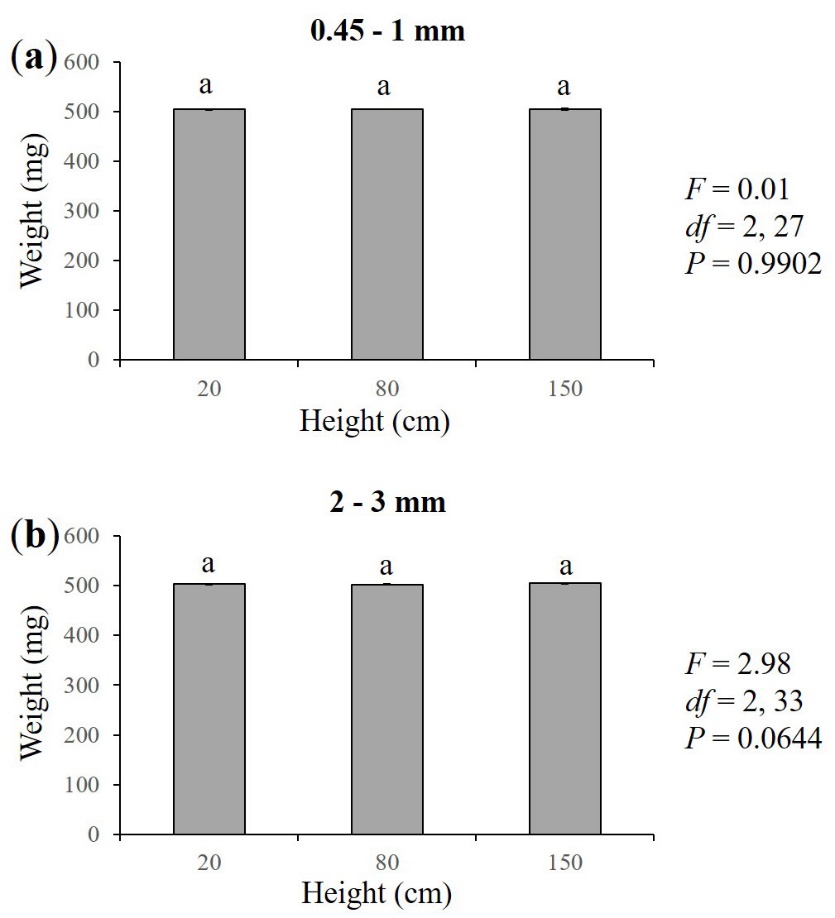


**Fig. S4:** Tubes pasted on the tree trunks at the height of 20, 80, or 150 cm contained similar weight of food with particle sizes ranging from (a) 0.45-1 mm, or (b) 2-3 mm. Data were compared using ANOVA. The same letter indicates no significant difference (*P* > 0.05).

**Table S1:** General information about each video that used to investigate the behavioral responses of *Solenopsis invicta* workers to food with different sizes.

| Tree No. | DBH ^a^ | Height | Food size | | | |
| --- | --- | --- | --- | --- | --- | --- |
|  |  |  | 1×1×1 mm | 3×3×1 mm | 5×5×1 mm | 8×8×1 mm |
| 1 | 16.3 cm | 118 cm | 27 October 2017 | 25 October 2017 | 26 October 2017 | 28 October 2017 |
| 2 | 29.0 cm | 122 cm | 31 October 2017 | 27 October 2017 | 29 October 2017 | 28 October 2017 |
| 3 | 26.3 cm | 118 cm | 25 October 2017 | 27 October 2017 | 29 October 2017 | 28 October 2017 |
| 4 | 19.9 cm | 102 cm | 28 October 2017 | 27 October 2017 | 25 October 2017 | 29 October 2017 |
| 5 | 20.3 cm | 101 cm | 3 November 2017 | 31 October 2017 | 1 November 2017 | 29 October 2017 |
| 6 | 36.0 cm | 124 cm | 29 October 2017 | 31 October 2017 | 3 November 2017 | 1 November 2017 |

^a^ DBH: Diameter at breast height
